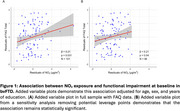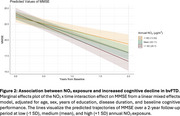# Exposure to ambient air pollution is associated with greater functional impairment and faster cognitive decline in behavioral‐variant frontotemporal degeneration

**DOI:** 10.1002/alz70860_107415

**Published:** 2025-12-23

**Authors:** Rory Boyle, Brian Nelson, Sheina Emrani, Katheryn A. Q. Cousins, Shana D. Stites, David J. Irwin, Corey T. McMillan, Lauren Massimo

**Affiliations:** ^1^ Department of Neurology, University of Pennsylvania Perelman School of Medicine, Philadelphia, PA, USA; ^2^ University of Pennsylvania, Philadelphia, PA, USA

## Abstract

**Background:**

Exposure to elevated levels of ambient air pollutants, including nitrogen dioxide (NO2), are associated with higher risk of all‐cause dementia [1‐3] as well as lower cognitive performance in community‐dwelling older adults [4]. However, it is unclear if exposure to NO2 is associated with cognitive and/or functional impairment and disease progression in behavioral‐variant frontotemporal degeneration (bvFTD).

**Method:**

Individuals with a primary clinical diagnosis of bvFTD from the Penn Frontotemporal Degeneration Center had available data for NO2 exposure and cognitive performance (*n* = 168) or NO2 exposure and functional impairment (*n* = 101). An annual average of census‐tract level exposure to NO2 was obtained for each individual from the Health and Retirement Study's Contextual Data Resource and matched to the census‐tracts of their home address. Cognitive performance was assessed using the Mini‐Mental State Examination (MMSE) at baseline and over a 2‐year follow‐up period. Functional impairment was assessed at baseline using the Functional Activities Questionnaire (FAQ). Separate linear regression models for baseline cognitive performance and functional impairment examined associations with NO2 exposure, adjusting for age, sex, years of education, and disease duration (time from symptom onset to initial assessment). Linear mixed effects models related NO2 exposure to change in cognitive performance over time, additionally covarying for baseline performance.

**Result:**

In linear regressions, NO2 exposure was associated with greater baseline functional impairment (β = 0.21, 95% CI = 0.02 to 0.41, *p* = 0.031, see Figure 1) but not with baseline cognitive performance (β = ‐0.09, 95% CI = ‐0.24 to 0.05, *p* = 0.212). In linear mixed‐effects models, NO2 exposure was associated with steeper global cognitive decline (NO2 x time β = ‐0.08, 95% CI = ‐0.13 to ‐0.02, *p* = 0.008, see Figure 2).

**Conclusion:**

Exposure to elevated NO2 is associated with worse functional impairment at baseline and faster subsequent cognitive decline. Imaging and pathologic studies are necessary to identify potential mechanisms of these associations.

**References**

[1]. Carey et al. 2014 BMJ Open https://doi.org/10.1136/bmjopen‐2018‐022404

[2]. Tang et al. 2023 Environmental Research https://doi.org/10.1016/j.envres.2022.115048

[3]. Wilker et al. 2023 BMJ https://doi.org/10.1136/bmj‐2022‐071620

[4]. Kulick et al. 2020 Neurology https://doi.org/10.1212/WNL.0000000000009314